# Hypertension and Obesity in Adults Living in a High HIV Prevalence Rural Area in South Africa

**DOI:** 10.1371/journal.pone.0047761

**Published:** 2012-10-17

**Authors:** Abraham Malaza, Joel Mossong, Till Bärnighausen, Marie-Louise Newell

**Affiliations:** 1 Africa Centre for Health and Population Studies, University of KwaZulu-Natal, Somkhele, KwaZulu-Natal, South Africa; 2 Department of Global Health and Population, Harvard School of Public Health, Harvard University, Boston, Massachusetts, United States of America; 3 Centre for Paediatric Epidemiology and Biostatistics, University College London Institute of Child Health, London, United Kingdom; University of Cape Town, South Africa

## Abstract

Hypertension and excess body weight are major risk factors of cardiovascular morbidity and mortality in developing countries. In countries with a high HIV prevalence, it is unknown how increased antiretroviral treatment and care (ART) coverage has affected the prevalence of overweight, obesity, and hypertension. We conducted a health survey in 2010 based on the WHO STEPwise approach in 14,198 adult resident participants of a demographic surveillance area in rural South Africa to investigate factors associated with hypertension and excess weight including HIV infection and ART status. Women had a significantly higher median body mass index (BMI) than men (26.4 vs. 21.2 kg/m^2^, p<0.001). The prevalence of obesity (BMI≥30 kg/m^2^) in women (31.3%, 95% confidence interval (CI) 30.2–32.4) was 6.5 times higher than in men (4.9%, 95% CI 4.1–5.7), whereas prevalence of hypertension (systolic or diastolic blood pressure≥140 or 90 mm Hg, respectively) was 1.4 times higher in women than in men (28.5% vs 20.8%, p<0.001). In multivariable regression analysis, both hypertension and obesity were significantly associated with sex, age, HIV and ART status. The BMI of women and men on ART was on average 3.8 (95% CI 3.2–3.8) and 1.7 (95% CI 0.9–2.5) kg/m^2^ lower than of HIV-negative women and men, respectively. The BMI of HIV-infected women and men not on ART was on average 1.2 (95% CI 0.8–1.6) and 0.4 (95% CI -0.1–0.9) kg/m^2^ lower than of HIV-negative women and men, respectively. Obesity was a bigger risk factor for hypertension in men (adjusted odds ratio (aOR) 2.99, 95% CI 2.00–4.48) than in women (aOR 1.64, 95% CI 1.39–1.92) and overweight (25≤BMI<30) was a significant risk factor for men only (aOR 1.53 95% CI 1.14–2.06). Our study suggests that, cardiovascular risk factors of hypertension and obesity differ substantially between women and men in rural South Africa.

## Introduction

At the beginning of the 20^th^ century, cardiovascular disease (CVD) was responsible for less than 10% of all deaths worldwide, but by 2008 that figure had risen to 30% [1]. About 80% of the global burden of CVD death occurs in low- and middle-income countries [2]. Historically, excess body weight and high blood pressure have been regarded as a ‘Western’ problem associated with affluence, but both are now also recognized as leading risk factors for cardiac diseases in low- and middle-income countries and have become important for public health globally [3], [4].

Reliable data on the main cardiovascular risk factors, obesity and hypertension, from low-and middle-income countries are scarce, especially from rural Africa [5]. In the Demographic Surveillance Area (DSA) covered by the surveillance system that is run by the Africa Centre for Health and Population Studies in rural KwaZulu-Natal, prevalence of obesity and hypertension among adults 15–50 year old was reported at 32% and 24% respectively in 2004, at the height of the HIV epidemic and before antiretroviral therapy (ART) was widely available in the public health service [5]. At that time, we hypothesized that HIV could “hide” the emerging epidemic of obesity, which has been recognized as a health risk also in South Africa (SA), and that this epidemic might be “unmasked” by large scale provision of ART in the community [6]. In the first nationally representative survey in SA with anthropometric data, mean body mass indices (BMI) in women and men were 27.1 and 22.9 kg/m^2^, respectively, with 56.6% of women and 29.2% of men overweight or obese [7]. Moreover, when using a cut-off point of 140/90 mm Hg, the hypertension prevalence was found to be 21% for both genders [8].

In addition to a high prevalence of obesity and hypertension, SA also has a high prevalence of HIV, and SA is home to the largest absolute number (estimated 5.6 million in 2009) of people living with HIV globally [9], [10]. In our setting in rural KwaZulu-Natal, HIV prevalence in 2011 was 24% among adults, with little evidence of declining incidence, which remains around 3% per year [11], [12]. Thus, like other countries in Southern Africa, SA is facing an increasing burden of both communicable (HIV and TB) and non-communicable (cardiovascular) diseases [13].

In recent years ART use in sub-Saharan Africa has expanded rapidly, with an estimated 3.9 million patients on treatment in 2009 [14]. SA has the largest public ART programme in the world [14]. Using the 2006 WHO guidelines (eligibility CD4<200 cells/µl) ART coverage in the eligible adult population of SA was estimated at 40% in mid-2008 [15] and 56% at the end of 2008 [14]. HIV-related mortality rates have decreased due to the wide spread uptake of ART [16], [17], which results in increasing HIV prevalence rates and an aging HIV infected population. While the benefits of ART have revolutionized the treatment and care of HIV-infected individuals, the impact of this on non-communicable diseases needs to be elucidated. It is well-established that ART can reverse HIV-related weight loss and wasting [18], but population-based measures of the effect of ART on obesity prevalence in sub-Saharan Africa are lacking. Further, in developed countries ART has been associated with an increased relative risk of cardiovascular disease [19]. In our setting, over the recent years, the cause-specific mortality fraction for non-communicable lifestyle-related conditions was 15% in the overall population; non-communicable disease mortality increased with age and was the major cause of death in the 65-and-older age group [17].

We here present the data on weight, height and blood pressure in adults participating in our individual health surveillance in 2010, and investigate the relationship of HIV infection and ART status with obesity and hypertension.

## Methods

We conducted a large population-based survey of body mass index (BMI) and blood pressure (BP) in rural Umkhanyakude district of KwaZulu-Natal, South Africa. The survey took place in 2010, as part of the longitudinal population-based HIV and Health surveillance conducted by the Africa Centre for Health and Population Studies [20]. Individuals are eligible for HIV surveillance if they are reported to be member of a household within a defined geographic demographic surveillance area (DSA) even if non-resident at the time of surveillance. Membership is self-defined on the basis of links to other household members and residency is based on residing at a physical structure within the surveillance area at a particular point in time. For this analysis, the study population consisted of adult (≥15 years) residents of the DSA.

Adults who participate in the surveillance were asked to provide a small finger prick blood sample for HIV infection status measurement. HIV status was assessed by enzyme-linked immunosorbent assay (ELISA) of EDTA anticoagulated blood samples in the Africa Centre virology laboratory, using HIV-1/HIV-2 ELISA assay (Vironostika; Organon Taknika, Boxtel, the Netherlands).

Height, weight and blood pressure (BP) measurements were taken following the WHO STEPS protocol [21]. When both weight and height measurements were taken, BMI was calculated as the weight in kilograms divided by the square of heights in meters [22]. The following BMI categories were used : underweight (BMI<18.5), normal weight (18.5≤BMI<25), overweight (25≤BMI<30) and obese (BMI≥30 kg/m^2^). The averages of the second and third diastolic blood pressure (dBP) and systolic blood pressure (sBP) measurements were used to estimate dBP and sBP, respectively. Hypertension was defined as sBP≥140 mm Hg or dBP≥90 mm Hg; stage-I hypertension (140≤sBP<160 mm Hg) and/or (90≤dBP<100 mm Hg) and stage-II hypertension was defined as sBP≥160 mm Hg and/or dBP≥100 mm Hg, as per guidelines of the European Society of Cardiology [23].

To investigate risk factors associated with BMI and hypertension we performed linear and logistic regression analyses, respectively. BMI and hypertension were independently regressed against previously investigated [5] explanatory variables age, sex, household asset index, education level, place of residence (rural, peri-urban, urban), as well as a combined HIV/ART status (HIV-negative; HIV-positive and initiated on ART, HIV-positive and not initiated on ART, unknown HIV/ART status). Although HIV status is measured annually, for this analysis HIV status was determined in the 2010 round visit during which weight, height and BP were measured. Weight was assessed as an independent variable to the regression with hypertension as it was previously found to be an independent risk factor [5]. Information on whether an HIV infected adult had been initiated on ART was obtained from the local district HIV treatment and care programme database which is housed at the Africa Centre, and which information can be linked to that of the surveillance at an individual level [24].

Demographic, social and economic data about survey participants were available from the Africa Centre household surveillance [20]. As a measure of household wealth, we used an asset index based on living conditions (household ownership, access to piped water, access to electricity, main energy source for cooking, toilet type) and 27 household assets [25]. Households were categorized into quintiles of the asset index at the DSA level.

For education, we used dummy variables corresponding to the categories of educational attainment in the South African education system: no schooling, primary school (grades 1–3), higher primary (grades 4–6), high school (grades 7–10) and tertiary (above grade 10). Data analysis and graphics were produced in STATA 11.0 (State Corporation, College Station, TX, USA). Approval for the household surveillance, HIV surveillance and linkage to the HIV treatment and care programme database was obtained from the Bio-Medical Ethics Committee of the University of KwaZulu-Natal.

## Results

**Figure 1:**
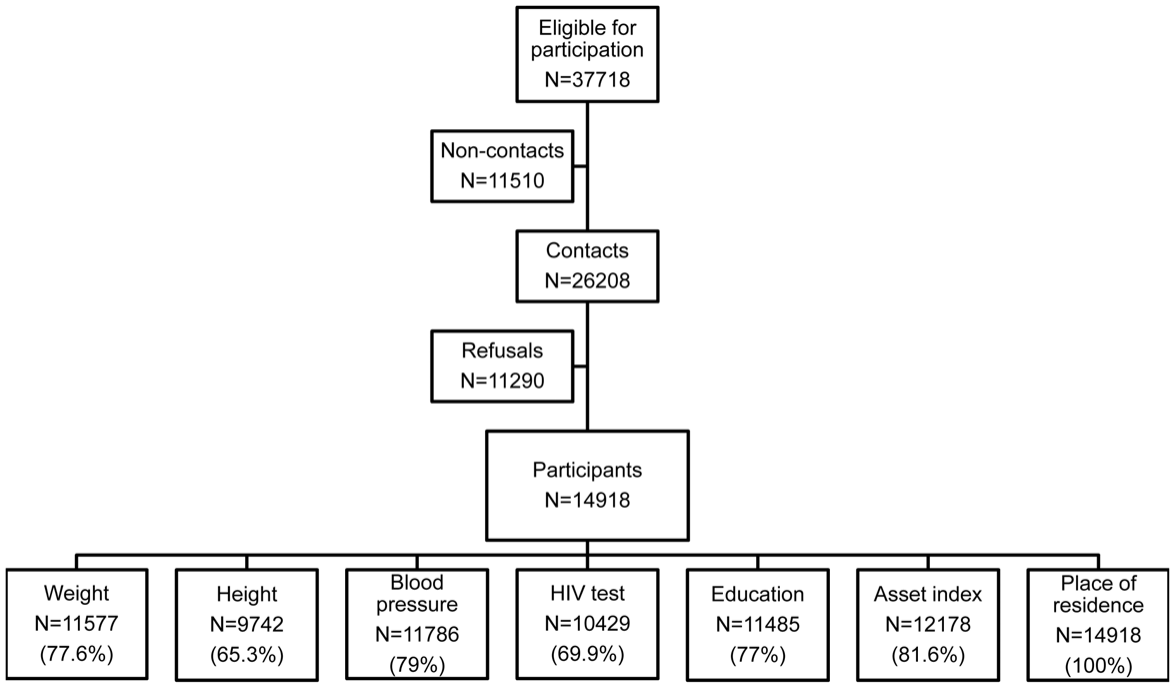
Flow diagram of individuals participating in the various aspects of the health and demographic surveillance. Non-contacts refer to eligible individuals who were not contacted due to death, illness or had out-migrated outside the demographic study area.

A total of 37,718 resident adults were identified from the household surveillance membership to be eligible to participate in the study, of whom 11,510 (31%) were not contactable, mainly due to migration, death or severe illness. Of the 26,208 (69%) contactable individuals, 14,918 (57%) agreed to participate in at least one component of the study. Data on weight, height, blood pressure, HIV status, education, asset index and place of residence were available in 78%, 65%, 79%, 70%, 77%, 82% and 100% of study participants, respectively. Figure 1 depicts the numbers of study participants consenting to the various components of the surveillance.

### Weight, height and BMI

**Figure 2:**
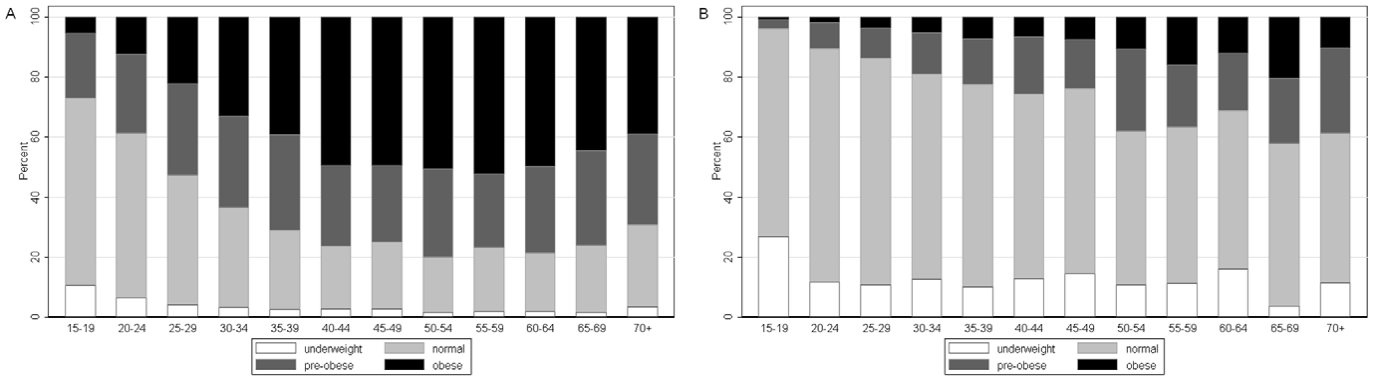
Distribution of body mass index by age group in (A) women and (B) men. BMI categories (underweight: less than 18.5 kg/m^2^; normal: between 18.5 and 25 kg/m^2^; overweight: between 25 and 30 kg/m^2^; obese: greater than 30 kg/m^2^) .

The median weight was 64.6 kg overall, with women having a significantly (p<0.001) higher (67.3 kg) median weight than men (61.3 kg). Men had a significantly (p<0.001) higher (1.70 m) median height than women (1.60 m) with an overall population median height of 1.62 m (Table 1). A total of 9,612 adults (6,688 women and 2,924 men) had both valid weight and height measurements. The median BMI was 24.2 kg/m^2^, with women having a significantly higher median BMI than men (26.4 vs. 21.2 kg/m^2^, p<0.001). The prevalence of overweight was 45.7% (95% confidence interval (CI) 44.7–46.7), 58.7% (95% CI 57.5–59.9) among women and 15.9% (95% CI 14.6–17.2) among men. The prevalence of overweight was 22.4% (95% CI 21.6–23.3), 27.4% (95% CI 26.3–28.5) among women and 11.0% (95% CI 9.9–12.2) among men. The prevalence of obesity was 23.3% (95% CI 22.4–24.1), 31.3% (95% CI 20.2–32.4) among women and 4.9% (95% CI 4.1–5.7) among men. Figure 2 shows two distinct sex-specific age patterns of BMI distributions. In women, obesity prevalence rises rapidly from rather low levels in early adulthood, reaches a plateau for women between the ages 40 and 65 years and then decreases again for older women. The proportion of overweight women (25≤BMI<30) changes very little with age. In men, the prevalence of overweight and obesity rises from very low levels gradually with age, although to a lower extent and without the plateauing and later decrease observed in women.

**Table 1:** Anthropometric measurements, body-mass index (BMI), blood pressure, and hypertension staging by HIV infection status and sex.

	Total		HIV uninfected		HIV infected	
	Women	Men	Women	Men	Women	Men
Weight (kg)^1^	67.3 (57.1−80.8)	61.3 (54.7−68.7)	65.8 (56.6−77.0)	61.9 (55.8−68.5)	67.5 (57.1−82.2)	60.5 (54.0−68.0)
Height (m)^1^	1.60 (1.55−1.64)	1.70 (1.65−1.74)	1.59 (1.55−1.63)	1.70 (1.65−1.74)	1.60 (1.56−1.65)	1.71 (1.66−1.76)
BMI (kg/m^2^)^1^	26.5 (22.5−31.6)	21.2 (19.2−23.6)	26.7 (22.6−32.2)	21.0 (19.0−23.5)	25.4 (22.1−29.8)	21.1 (19.5−23.6)
sBP (mm Hg)^1,2^	117 (107−134.5)	119.5 (111.5−131)	119.5 (108−138.5)	120 (111.5−132)	113 (104.5−125)	119.5 (111−128.5)
dBP (mm Hg)^1,2^	80 (72.0−89.5)	76.5 (69−85)	80.5 (72−90.5)	76 (68.5−84.5)	78 (71−86.5)	78 (70−85)
Underweight (BMI<18.5)	311 (4.7%)	495 (16.9%)	191 (4.8%)	370 (18.6%)	86 (5.5%)	53 (14.4%)
Normal (18.5≤BMI<25)	2451 (36.7%)	1964 (67.2%)	1391 (34.8%)	1312 (65.9%)	653 (41.4%)	259 (70.2%)
Overweight (25≤BMI<30)	1833 (27.4%)	323 (11.0%)	1058 (26.4%)	207 (10.4%)	462 (29.3%)	42 (11.4%)
Obese (BMI≥30)	2093 (31.3%)	142 (4.9%)	1365 (34.1%)	102 (5.1%)	375 (23.8%)	15 (4.1%)
Stage−I hypertension^3^	1244 (15%)	439 (12.5%)	763 (15.9%)	268 (11.8%)	210 (11.7%)	52 (11.7%)
Stage-II hypertension^4^	1118 (13.5%)	289 (8.3%)	744 (15.5%)	201 (8.9%)	151 (8.4%)	24 (5.4%)

1 Median (interquartile range)

2 sBP (systolic blood pressure), dBP (diastolic blood pressure)

3 Stage-I hypertension refers to systolic sBP between 140–160 mmHg and/or dBP between 90–100 mmHg.

4 Stage-II hypertension refers to sBP greater 160 mmHg and/or dBP greater than 100 mmHg.

### Blood pressure

**Figure 3:**
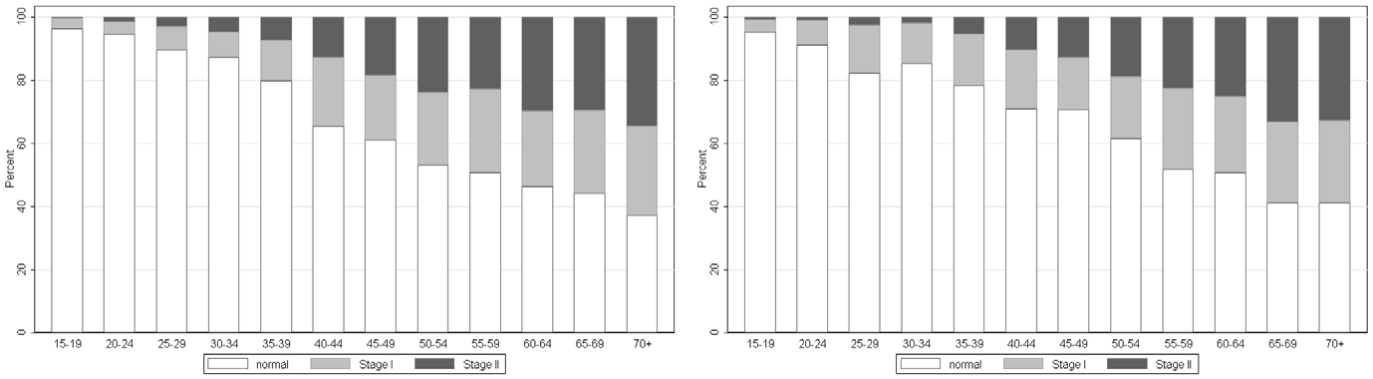
Distribution of hypertension by age group in (A) women and (B) men. Hypertension categories (normal: systolic BP less 120 or diastolic BP less 80 mmHg; stage-I hypertension: systolic BP between 140–160 mmHg and/or diastolic BP between 90–100 mmHg; stage-II hypertension: systolic BP greater 160 mmHg and/or diastolic BP greater than 100 mmHg).

The overall median for sBP was 118 mm Hg, men had a median of 119.5 mm Hg and women had a median of 117 mm Hg (p<0.001). Median dBP was 78.5 mm Hg overall, 80 mm Hg for women and 76.5 for men (p<0.001). Women had a higher prevalence of hypertension than men (28.5% vs. 20.8%, p<0.001). The prevalence of stage-I and stage-II hypertension was 14.3% (95% CI 13.7–14.9) and 11.9% (95% CI 11.4–12.5), respectively. Among women, 15% (95% CI 14.3–15.8) had stage-I hypertension and 13.5% (95% CI 12.8–14.3) had stage-II hypertension and among men, 12.5% (95% CI 11.4–13.7) had stage-I hypertension and 8.3% (95% CI 7.4–9.2) stage-II hypertension. Figure 3 shows that the prevalence of both stage-I and stage-II hypertension increase almost linearly with age for both sexes.

### HIV status, BMI and hypertension

Among the 10,429 participants with valid HIV results, overall HIV prevalence was estimated at 24.1% (95% CI; 23.3–25.0%). Women were significantly more likely to be HIV infected than men (27.6% vs. 16.2%, p<0.001). The prevalence of overweight did not differ significantly between HIV-infected and HIV-uninfected adults (45.6 vs. 46.0%, p = 0.76), but overall obesity was significantly more common among HIV-uninfected than infected adults (20.0% vs. 24.5%, p<0.001); this difference was driven by women (34% in HIV-uninfected vs. 23.8% in HIV infected women, p<0.001); while in men, there was no significant association between obesity and HIV-status (p = 0.39).

Prevalence of hypertension differed between HIV-infected and HIV-uninfected individuals (19.5% vs. 27.9%, p<0.001). HIV-uninfected women were significantly more likely to be hypertensive than HIV-infected women (31.4% vs. 20.1%, p<0.001) while there was no association of hypertension with HIV-status among men (p = 0.099). Table 1 shows the prevalence of stage-I and stage-II hypertension and mean BMI, dBP, sBP for all study participants, HIV-infected and HIV-uninfected individuals stratified by sex and age groups.

### Factors associated with BMI

In univariate regression analysis, BMI was independently associated with sex, age, wealth, educational attainment, HIV and ART status, but not place of residence (see table S1). When stratifying by sex, BMI was significantly associated with HIV and ART status among women, but not among men. For both women and men, there was an increasing trend of BMI with wealth, but not with educational attainment.

In multivariate regression analysis including both sexes, BMI remained independently associated with sex, age, wealth, educational attainment, HIV and ART status, but not place of residence (Table S1). Stratifying the analysis by sex showed that the increase of BMI with age in women was approximately twice that in men. The association of BMI with HIV and ART-status was also more pronounced in women: the BMI of women and men on ART was on average 3.8 (95% CI 3.2–3.8) and 1.7 (95% CI 0.9–2.5) kg/m^2^ lower than of HIV-negative women and men, respectively. The BMI of HIV infected women not on ART was on average 1.2 (95% CI 0.8–1.6) kg/m^2^ lower than of HIV-negative women. The BMI of HIV positive men not on ART was not statistically different from HIV-negative men (p = 0.15). For both men and women, BMI increased with wealth, which was more pronounced in women than men. BMI had an increasing trend with educational attainment in men, but not in women. For both men and women, BMI was not associated with place of residence.

### Factors associated with hypertension

In univariate analysis, all explanatory variables (sex, age, HIV/ART status, wealth, place of residence area, educational attainment and BMI) were significantly associated with hypertension (Table S2). In multivariable analysis, sex, age, HIV and ART-status, BMI, place of residence, educational attainment remained significantly associated with hypertension, but not sex and wealth. The adjusted odds ratio of hypertension increased by 5.3% (95% CI 5.0–5.7) per age year. HIV infected persons on ART treatment had a reduced adjusted odds ratio of hypertension (aOR 0.60, 95% CI 0.49–0.75), compared to HIV-negative persons. The adjusted odds ratio in HIV infected persons not on ART did not differ from HIV-negative persons (p = 0. 0.158). Stratifying the analysis by sex showed that obesity was a bigger risk factor for men (aOR 2.99, 95% CI 2.00–4.48) than for women (aOR 1.64, 95% CI 1.39–1.92) and that overweight was a significant risk factor for men (aOR 1.53 95% CI 1.14–2.06), but not for women (aOR 1.14 95% CI 0.97–1.36), while controlling for other factors.

## Discussion

The high prevalence of obesity and overweight is indicative of the epidemiological transition from diseases of poverty to those of affluence in this rural South African community. Our study confirms previous findings of obesity and hypertension disproportionally affecting women, especially at younger ages, in South Africa [5], [7], [13], and other sub-Saharan countries [26], [27].

The reasons behind the local obesity patterns are likely to be multi-factorial. It has been observed that in low- and middle-income countries, the burden of obesity has shifted to low socioeconomic status groups and rural areas as a country's gross domestic product (GDP) increases and that the highest prevalence of overweight and obesity are in middle-age groups (45–59 years) throughout this transition [28]. Possible structural risk factors for obesity include subsidized agriculture and multinational companies providing cheap, highly refined fats, oils, and carbohydrates, labour-saving mechanized devices, affordable motorized transport, and sedentary behaviour [26]. In addition to the above global environmental trends, there also local socio-behavioural contributing factors. Ethnic black overweight and obese women often do not view themselves as overweight. Moderately overweight women are perceived by the community as attractive, as this is associated with respect, dignity and affluence [29]. In areas of high HIV prevalence, thinness is often associated with HIV [29].

Our study agrees with previous findings from other low and middle-income countries that obesity in both women and and men was associated with higher socioeconomic status, whereas in developed countries obesity was associated with poverty [30], [31]This is likely due the fact that we are using a relative conception of wealth in this study (wealth quintiles) and that absolute poverty and unemployment levels are very high in this community, with government grants frequently being the only source of income.

Our study is one of the first in Southern Africa to assess the association of widespread ART with obesity and hypertension at a population level. HIV prevalence in this community continues to be high (24% in 2010) and our study confirms previous findings that HIV-infection is associated with lower BMI and thus a lower prevalence of obesity and overweight [5]. In addition, our study suggests that this effect is more pronounced in women than in men: women on ART had 3.9 units of BMI lower compared to 1.8 units in men, when compared to their respective counter parts who are HIV-uninfected. Moreover, while we would expect ART to be associated with larger body mass, we actually found the opposite: while controlling for factors including age, sex and socioeconomic status, HIV-infected individuals on ART had a lower BMI than HIV-infected individuals not on ART. The negative association of of ART with BMI in HIV infected individuals could be attributed to late presentation and initiation of individuals on ART [24], the short duration of treatment or the disproportional numbers between women and men in the ART programme, and a restrictive eligibility criterion (CD4<200 cells/µl) at the time of the study. Thus ART patients are likely to be in a more advanced stages of HIV disease (relatively low CD4 counts at ART initiation), resulting in more severe weight loss than HIV-infected patients not on ART. The fact that we could not adjust for disease stage and treatment duration is clearly an important study limitation. It will be interesting to conduct further studies in the future to assess whether long durations of ART treatment are associated with metabolic syndrome.

Although a direct biological link is likely, it is also possible that HIV-infected persons are more in contact with health care services, so they may be more susceptible to adopt nutrition-related advice. Furthermore, while ART drugs are given free of charge, travel costs to receive ART are not reimbursed, thus patients on ART in this poor community have a lower budget left for food [32]. Further research in our longitudinal demographic and HIV surveillance should be conducted to investigate the main risk factors of lower BMI in adults receiving ART.

Although we found that hypertension was clearly associated with known risk factors like age, we observed that independent association of excessive weight with hypertension was stronger in men than in women. Overweight (25≤BMI<30) men but not women were at increased risk of hypertension, after controlling for other factors. Similar sex differences were observed in an Eritrean study where the effect of BMI on blood pressure was higher in males than in females [33]. The absence of an independent positive association between excess weight and hypertension has also been reported before in South Africa [34], [35], although the ‘benign obesity’ concept developed has been questioned [29]. One of the limitations of our study was that we did not to measure other indicators of metabolic syndrome including waist circumference, cholesterol, triglycerides, insulin resistance, nor well known risk factors like smoking that may lead to heart disease and diabetes. The role of obesity and overweight as independent risk factors for cardiovascular disease is still debated in developed countries: a French study found that overweight subjects without associated risk factors (hypertension or hypercholesterolemia) did not have an increased risk of cardiovascular mortality suggesting that the presence of high blood pressure in overweight subjects might be a key factor [36].

While our study highlights the high prevalence of obesity in this limited resources rural setting, it is less obvious which, if any, interventions can actually help to combat this situation, as almost all efforts to lose weight, in the long-term have resulted in failure, or in very short-term success [37]. Obesity will not be prevented simply by telling individuals and communities to change their diet and increase exercise [22]. The benefits and costs of any intervention should thus to be carefully balanced against potentially competing public health needs in areas with very high HIV prevalence, where the provision of ARVs have unquestionably lead to substantial morbidity and mortality reductions.

## Supporting Information

Table S1 **Univariate and multivariate least square regression coefficients (standard errors) relating body mass index (BMI) to explanatory variables.**
(DOC)

Table S2 **Univariate and multivariate logistic regression odds ratios (standard errors) relating hypertension to explanatory variables.**
(DOC)
