# Supplementary material for: Hypertension and Obesity in Adults Living in a High HIV Prevalence Rural Area in South Africa
Source: PLoS One. 2012 Oct 17;7(10):e47761. doi: 10.1371/journal.pone.0047761 (PMC3474786; doi:10.1371/journal.pone.0047761)
Supplement: Table S1 — Univariate and multivariate least square regression coefficients (standard errors) relating body mass index (BMI) to explanatory variables. (DOC) [file pone.0047761.s001.doc]

**Table S1: Univariate and multivariate least square regression coefficients (standard errors) relating body mass index (BMI) to explanatory variables.**

|  | Both sexes | | Women | | Men | |
| --- | --- | --- | --- | --- | --- | --- |
|  | Univariate | Multivariate | Univariate | Multivariate | Univariate | Multivariate |
| Sex |  |  |  |  |  |  |
| Male | Ref. | Ref. |  |  |  |  |
| Female | 5.62 (0.132)*** | 4.680 (0.123)*** |  |  |  |  |
| Age group |  |  |  |  |  |  |
| 15-24 | Ref. | Ref. | Ref. | Ref. | Ref. | Ref. |
| 25-34 | 3.23 (0.174)*** | 2.981 (0.169)*** | 3.236 (0.217)*** | 3.826 (0.222)*** | 1.373 (0.211)*** | 1.391 (0.218)*** |
| 35-44 | 5.75 (0.197)*** | 5.465 (0.195)*** | 5.989 (0.238)*** | 6.613 (0.250)*** | 2.122 (0.261)*** | 2.534 (0.278)*** |
| 45-54 | 6.833 (0.190)*** | 6.228 (0.199)*** | 6.924 (0.227)*** | 7.271 (0.255)*** | 2.646 (0.269)*** | 3.260 (0.290)*** |
| 55-64 | 6.577 (0.217)*** | 6.020 (0.226)*** | 6.825 (0.261)*** | 7.007 (0.293)*** | 3.142 (0.287)*** | 3.796 (0.314)*** |
| 65-74 | 6.296 (0.249)*** | 5.461 (0.258)*** | 6.179 (0.297)*** | 6.111 (0.332)*** | 3.950 (0.339)*** | 4.431 (0.361)*** |
| 75+ | 4.516 (0.301)*** | 3.728 (0.308)*** | 4.094 (0.355)*** | 4.141 (0.390)*** | 3.193 (0.417)*** | 3.702 (0.443)*** |
| HIV/ART status |  |  |  |  |  |  |
| HIV-negative | Ref. | Ref. | Ref. | Ref. | Ref. | Ref. |
| HIV+ not on ART | 0.0531 (0.194) | -1.116 (0.170)*** | -1.045 (0.223)*** | -1.195 (0.210)*** | 0.283 (0.268) | -0.386 (0.266) |
| HIV+ on ART | -0.908 (0.282)** | -3.382 (0.243)*** | -2.134 (0.319)*** | -3.761 (0.297)*** | -0.348 (0.407) | -1.708 (0.394)*** |
| unknown | 0.0218 (0.179) | 0.0404 (0.150) | -0.102 (0.223) | -0.180 (0.201) | 0.363 (0.195) | 0.424 (0.185)* |
| Wealth Index |  |  |  |  |  |  |
| Poorest quintile | Ref. | Ref. | Ref. | Ref. | Ref. | Ref. |
| 2^nd^ quintile | 0.502 (0.222)* | 0.944 (0.186)*** | 0.881 (0.268)** | 1.068 (0.240)*** | 0.0646 (0.260) | 0.377 (0.247) |
| 3^rd^ quintile | 0.804 (0.220)*** | 1.383 (0.189)*** | 1.137 (0.263)*** | 1.557 (0.243)*** | 0.192 (0.260) | 0.526 (0.254)* |
| 4^th^ quintile | 1.221 (0.222)*** | 1.915 (0.194)*** | 1.746 (0.269)*** | 2.171 (0.252)*** | 0.562 (0.259)* | 0.866 (0.257)*** |
| Richest quintile | 1.522 (0.249)*** | 2.194 (0.225)*** | 1.896 (0.303)*** | 2.235 (0.296)*** | 1.463 (0.284)*** | 1.544 (0.289)*** |
| unknown | 0.515 (0.219)* | 1.108 (0.226)*** | 0.839 (0.267)** | 1.032 (0.298)*** | 0.744 (0.251)** | 0.972 (0.286)*** |
| Place of residence |  |  |  |  |  |  |
| rural | Ref. | Ref. | Ref. | Ref. | Ref. | Ref. |
| peri-urban | -0.267 (0.145) | -0.153 (0.129) | 0.0316 (0.179) | -0.164 (0.171) | 0.00010 (0.161) | -0.0661 (0.163) |
| urban | -0.0125 (0.295) | -0.490 (0.257) | -0.344 (0.356) | -0.700 (0.335)* | 0.861 (0.344)* | 0.351 (0.336) |
| Education |  |  |  |  |  |  |
| none | Ref. | Ref. | Ref. | Ref. | Ref. | Ref. |
| primary | -0.147 (0.306) | 0.0898 (0.263) | 0.146 (0.348) | 0.0221 (0.320) | -0.748 (0.436) | 0.197 (0.422) |
| higher primary | -1.833 (0.276)*** | 0.739 (0.250)** | -0.288 (0.331) | 0.910 (0.316)** | -2.030 (0.351)*** | 0.271 (0.366) |
| high school | -3.686 (0.211)*** | 0.395 (0.224) | -3.341 (0.244)*** | 0.296 (0.283) | -1.673 (0.295)*** | 0.893 (0.334)** |
| tertiary | -0.782 (0.374)* | 0.928 (0.345)** | -0.585 (0.444) | 0.773 (0.442) | 0.861 (0.470) | 1.949 (0.480)*** |
| unknown | -1.516 (0.231)*** | 0.617 (0.238)** | -0.848 (0.268)** | 0.780 (0.301)** | -0.662 (0.315)* | 0.525 (0.347) |

Abbreviations: Ref. – reference, *** p<0.001, ** p<0.01, * p<0.05
