# Supplementary material for: Hypertension and Obesity in Adults Living in a High HIV Prevalence Rural Area in South Africa
Source: PLoS One. 2012 Oct 17;7(10):e47761. doi: 10.1371/journal.pone.0047761 (PMC3474786; doi:10.1371/journal.pone.0047761)
Supplement: Table S2 — Univariate and multivariate logistic regression odds ratios (standard errors) relating hypertension to explanatory variables. (DOC) [file pone.0047761.s002.doc]

**Table S2: Univariate and multivariate logistic regression odds ratios (standard errors) relating hypertension to explanatory variables.**

|  | Both sexes | | Women | | Men | |
| --- | --- | --- | --- | --- | --- | --- |
|  | Univariate | Multivariate | Univariate | Multivariate | Univariate | Multivariate |
| Sex |  |  |  |  |  |  |
| Male | 1.0 | 1.0 |  |  |  |  |
| Female | 1.522 (0.0482)*** | 0.880 (0.0599)* |  |  |  |  |
| Age group |  |  |  |  |  |  |
| 15-24 | 1.0 | 1.0 | 1.0 | 1.0 | 1.0 | 1.0 |
| 25-34 | 2.621 (0.0982)*** | 2.482 (0.102)*** | 2.694 (0.131)*** | 2.519 (0.134)*** | 2.954 (0.155)*** | 2.808 (0.162)*** |
| 35-44 | 6.478 (0.0920)*** | 5.559 (0.0995)*** | 7.771 (0.120)*** | 6.439 (0.129)*** | 4.983 (0.160)*** | 4.914 (0.175)*** |
| 45-54 | 12.41 (0.0862)*** | 9.697 (0.0977)*** | 15.58 (0.114)*** | 11.54 (0.127)*** | 7.603 (0.156)*** | 7.376 (0.176)*** |
| 55-64 | 18.30 (0.0906)*** | 13.86 (0.103)*** | 21.97 (0.119)*** | 15.88 (0.133)*** | 14.06 (0.155)*** | 12.86 (0.180)*** |
| 65-74 | 25.32 (0.0973)*** | 18.79 (0.110)*** | 29.62 (0.126)*** | 21.24 (0.141)*** | 21.22 (0.171)*** | 18.01 (0.197)*** |
| 75+ | 29.99 (0.106)*** | 22.84 (0.120)*** | 36.70 (0.134)*** | 26.74 (0.150)*** | 21.25 (0.199)*** | 18.55 (0.228)*** |
| HIV/ART status |  |  |  |  |  |  |
| HIV-negative | 1.0 | 1.0 | 1.0 | 1.0 | 1.0 | 1.0 |
| HIV+ not on ART | 0.628 (0.0688)*** | 0.894 (0.0795) | 0.557 (0.0766)*** | 1.000 (0.0904) | 0.766 (0.160) | 0.642 (0.179)* |
| HIV+ on ART | 0.619 (0.102)*** | 0.603 (0.110)*** | 0.529 (0.113)*** | 0.652 (0.125)*** | 0.882 (0.232) | 0.478 (0.247)** |
| unknown | 0.973 (0.0523) | 1.060 (0.0623) | 0.903 (0.0619) | 0.997 (0.0732) | 1.174 (0.0991) | 1.209 (0.120) |
| Wealth Index |  |  |  |  |  |  |
| Poorest quintile | 1.0 | 1.0 | 1.0 | 1.0 | 1.0 | 1.0 |
| 2^nd^ quintile | 0.947 (0.0693) | 1.026 (0.0799) | 0.983 (0.0796) | 0.995 (0.0920) | 0.903 (0.144) | 1.144 (0.165) |
| 3^rd^ quintile | 0.897 (0.0686) | 1.003 (0.0816) | 0.902 (0.0787) | 0.965 (0.0937) | 0.918 (0.142) | 1.125 (0.170) |
| 4^th^ quintile | 0.802 (0.0711)** | 0.953 (0.0858) | 0.803 (0.0818)** | 0.908 (0.0989) | 0.841 (0.146) | 1.105 (0.176) |
| Richest quintile | 0.775 (0.0793)** | 0.855 (0.0997) | 0.731 (0.0929)*** | 0.761 (0.116)* | 0.970 (0.155) | 1.171 (0.197) |
| unknown | 0.882 (0.0690) | 0.980 (0.0960) | 0.887 (0.0804) | 0.973 (0.113) | 0.957 (0.138) | 1.024 (0.187) |
| Place of residence |  |  |  |  |  |  |
| rural | 1.0 | 1.0 | 1.0 | 1.0 | 1.0 | 1.0 |
| peri-urban | 0.895 (0.0461)* | 1.158 (0.0567)** | 0.920 (0.0541) | 1.163 (0.0666)* | 0.886 (0.0897) | 1.114 (0.111) |
| urban | 0.643 (0.105)*** | 0.899 (0.122) | 0.644 (0.121)*** | 0.972 (0.142) | 0.649 (0.212)* | 0.680 (0.247) |
| Education |  |  |  |  |  |  |
| none | 1.0 | 1.0 | 1.0 | 1.0 | 1.0 | 1.0 |
| primary | 0.760 (0.0837)** | 1.070 (0.0899) | 0.827 (0.0930)* | 1.106 (0.0990) | 0.544 (0.199)** | 0.878 (0.221) |
| higher primary | 0.410 (0.0814)*** | 1.038 (0.0929) | 0.488 (0.0936)*** | 1.005 (0.105) | 0.303 (0.174)*** | 1.178 (0.204) |
| high school | 0.135 (0.0668)*** | 0.845 (0.0869) | 0.130 (0.0774)*** | 0.794 (0.100)* | 0.167 (0.141)*** | 1.047 (0.183) |
| tertiary | 0.331 (0.116)*** | 1.058 (0.137) | 0.259 (0.142)*** | 0.863 (0.165) | 0.640 (0.215)* | 1.848 (0.261)* |
| unknown | 0.482 (0.0647)*** | 1.093 (0.0856) | 0.498 (0.0738)*** | 1.077 (0.0988) | 0.500 (0.140)*** | 1.221 (0.179) |
| Weight |  |  |  |  |  |  |
| normal | 1.0 | 1.0 | 1.0 | 1.0 | 1.0 | 1.0 |
| pre-obese | 2.065 (0.0635)*** | 1.264 (0.0738)** | 1.804 (0.0760)*** | 1.145 (0.0868) | 3.155 (0.135)*** | 1.531 (0.152)** |
| obese | 3.987 (0.0589)*** | 1.826 (0.0718)*** | 3.595 (0.0696)*** | 1.635 (0.0816)*** | 6.883 (0.181)*** | 2.994 (0.205)*** |
| unknown | 3.323 (0.0578)*** | 1.689 (0.0685)*** | 3.325 (0.0726)*** | 1.570 (0.0855)*** | 3.025 (0.0998)*** | 1.818 (0.116)*** |

Abbreviations: *** p<0.001, ** p<0.01, * p<0.05
